# Comparing the effect of independent and combined interventions of household chlorination and handwashing on diarrhea of under-fives in rural Dire Dawa, Eastern Ethiopia: a cluster randomized controlled trial

**DOI:** 10.11604/pamj.2021.40.239.29785

**Published:** 2021-12-20

**Authors:** Ephrem Tefera Solomon, Sirak Robele Gari, Helmut Kloos, Bezatu Mengistie Alemu

**Affiliations:** 1 Ethiopian Institute of Water Resources, Addis Ababa University, Addis Ababa, Ethiopia,; 2 University of California, San Francisco Medical Center, San Francisco, California, United States of America,; 3 Haramaya University, College of Health and Medical Sciences, Harar, Ethiopia

**Keywords:** Cluster randomized controlled trial, combined intervention, diarrhea, Ethiopia, independent intervention, under-five children

## Abstract

**Introduction:**

in poorly developed countries, no single intervention is adequate to interrupt diarrhea occurrence in rural households. However, the effect sizes of multiple interventions and participants combined adherence to the interventions are understudied. This study aimed at comparing combined intervention of water sanitation and hygiene (WASH) with each individual intervention in reducing diarrhea among under-five children in rural Dire Dawa.

**Methods:**

a cluster randomized controlled factorial trial was conducted between October 2018 and January 2019. Householders in the first, second and third arms received waterguard, soap and both, respectively. However, householders in the control arm were followed with their customary practices. Generalized estimation equations (GEE) with log link Poisson distribution was used to compute adjusted incidence rate ratio and the corresponding 95% CIs.

**Results:**

overall, 36% (aIRR = 0.64, 95% CI: 0.57 - 0.73), 41% (aIRR = 0.588, 95% CI: 0.53 - 0.65), and 41% (aIRR = 0.585, 95% CI: 0.53 - 0.65) reduction in incidence of diarrhea was observed in the water treatment, handwashing and combined arms, respectively. This study showed no additional benefit of combining the two interventions than the individual intervention.

**Conclusion:**

we recommend implementing either household water disinfection using sodium hypochlorite or household handwashing with hand hygiene promotion independently at large scale to vulnerable population to reduce diarrheal morbidity.

## Introduction

Diarrheal disease is the second leading cause of death in children under five years of age, killing around 525,000 under-five children annually [1]. The global millennium development goals (MDG) target for drinking water was met in 2010. In 2015, 6.6 billion people used an improved drinking water source. Nevertheless, 663 million people still lack improved drinking water sources and 2.4 billion people lack improved sanitation facilities. Eight out of 10 people without improved drinking water sources live in rural areas [2]. The World Health Organization (2016) targeted in its sixth sustainable development goal (SDG) the availability and sustainable management of water and sanitation for all by 2030. Hence, reductions in morbidity and mortality from infectious diarrheal diseases require improvements in the quality of water and general personal and environmental hygiene [3]. Household handwashing reduces contamination of hands, food, water, and fomites [4] and diarrhea among infants [5]. Recent studies on handwashing in Ethiopia reported that most important recommended times in preventing acute diarrhea were before preparing food and after defecation; on the other hand, most mothers who were knowledgeable about handwashing were not executing it accurately [6,7].

Interventions to improve water quality at the household level are more effective than those at the source [8]. Impacts of each components of water sanitation and hygiene (WASH) on diarrheal risk reduction were estimated to be 48% for handwashing with soap, 17% for improved water quality and 36% for excreta disposal [9]. Although, it has been said that interventions that seek to substantially reduce childhood diarrhea should consider an integrated approach [10], the available studies showed no additional benefit of combining household water disinfection by chlorination and handwashing in comparison to the independent interventions [11-13]. Nevertheless, their combination had better effect than the individual one of each in reducing diarrhea in adults [10]. However, little is known about the effect size of two or more interventions using factorial design in comparison to the independent interventions, and about the participants combined adherence to the interventions. Moreover, it is uncertain that whether the combined intervention do better than the independent interventions in reducing diarrheal incidence. Hence, this study tries to compare the combined intervention against the single interventions after employing compliance measurement for the interventions. This study aimed at: exploring the effectiveness of independent household water treatment and handwashing in reducing childhood diarrhea and whether their combination has a synergistic effect compared to individual interventions along with compliance measurements among children under the age of five years in rural areas of Dire Dawa Administration, Ethiopia.

## Methods

**Study area:** Dire Dawa is Ethiopia's second city administration in addition to Addis Ababa. Dire Dawa is located about 505km east of Addis Ababa. The administration consists of nine urban and 38 rural *kebeles* (the smallest administrative unit in Ethiopia) in an estimated area of 1,288 square kilometres The administration is characterized by a semi-arid climate with low and erratic bimodal rainfall with about 677mm annually. In the rural area, the minimum and maximum average monthly temperatures are 19.1^o^C and 32.4^o^C. Springs, shallow well, and deep well are the main drinking water sources in the rural areas supplying 71.8% of the households in 2017. Seven health centers and 33 health posts exist in the rural area. According to the Central Statistical Agency report in 2013, the projected population of Dire Dawa in 2018 was 479,000 of which 240,000 were males and 239,000 were females. Of these, 176,000 (36.7%) lived in rural areas. The four rural districts had 34,150 households and 20,118 under-five children. Hence, an average of 6.8 persons was living in each household in the rural area.

**Source and study population:** all households having at least one under-five child in the 38 rural *kebeles* of the four districts were the source population and the under-five children in the randomly selected four *kebeles* were the study populations.

**Inclusion and exclusion criteria:** children aged from six through 59 months were included. The excluded households were those having severely ill mothers/caregivers, children aged less than six months, and children with persistent diarrhea.

**Figure 1:**
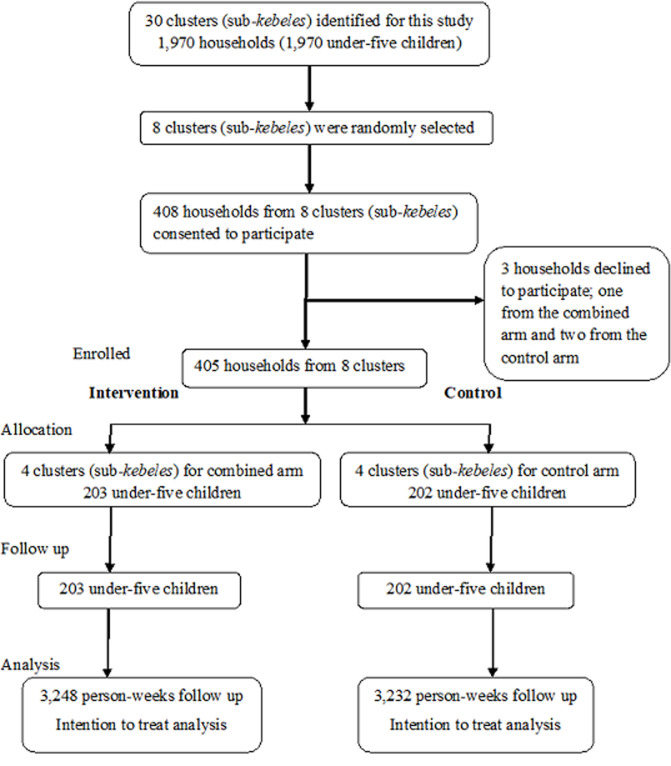
study participant selection and follow up flow for the community randomized controlled trial in the combined water treatment and handwashing arm versus control arm, rural Dire Dawa, Eastern Ethiopia, 2019

**Study design and procedure:** a cluster randomized controlled trial was conducted from October 2018 through January 2019 to evaluate the effectiveness of individual and combined interventions in reducing episodes of diarrhea among under-five children. The interventions were disinfection of household drinking water by chlorination, and handwashing, and the combination of the two. The 38 *kebeles* in rural Dire Dawa are located in 4 districts (locally known as Wereda which is the third level administrative division below Region and Zone in Ethiopia). Each kebele was divided into sub-*kebeles* (clusters) for the present study (clusters defined as having distinct neighborhoods with defined geographical boundaries). From the four districts, nine *kebeles* consisting of 55 sub-*kebeles* were identified. Four *kebeles* were selected from 38 *kebeles* using simple random sampling. Finally, the intervention studies were conducted in 16 sub-*kebeles* selected randomly within four *kebeles*. Eligible households for this study were those households with at least one under-five child. In households with more than one under-five child; the index child was selected by the lottery method. Figure 1 showed participant selection procedure from the combined and control arms. We calculated incidence of diarrhea as the number of new diarrhea episodes per person-time (i.e. person-weeks observation) [14]. Information on episodes of diarrhea was collected for 16 weeks during eight interviews once every two weeks.

The recruited clusters were randomized to the water treatment (WT), handwashing (HW), combined water treatment and handwashing (Co) and control (C) arms using the lottery method by the study team in the presence of the community leaders. The randomization was carried out by labeling each cluster using a unique number on piece of papers which were matched with equal number of papers labeled with 'WT', 'HW', 'Co' and 'C' by randomly picking from the containers by two non-field workers. In the present study, cluster randomization is primarily promoted to minimize treatment “contamination” among participants of the study arms [15]. Moreover, the intervention and control clusters were sufficiently far apart to reduce the possibility of information contamination between the clusters. After conducting a baseline survey, the intervention materials bottles of “waterguard” (one bottle for a month) and bars of soaps (two bars for 14 days) were distributed at a household level to each household in the intervention (WT, HW, and Co) arms. The intervention households were visited by the intervention providers once every two weeks to encourage them to internalize the new behavior of treating drinking water with chlorine as well as handwashing.

**Intervention:** the interventions were: household chlorination using waterguard, handwashing with soap at five critical times (before preparation of food, before intake of food, before feeding the child, after toilet use and after removal of child feces) and the combination of the two. The intervention households were coached by intervention providers to properly use the intervention materials. The intervention materials; waterguard and soap were provided to the intervention households free of charge throughout the study period. The handwashing facilities available in most study households were buckets with taps. However, participants in the control arm were followed with their usual habits of drinking water collection, water handling, water storage and handwashing practices. The trial adhered to Consort guidelines and a separate Consort checklist was prepared. Waterguard is the local brand name of sodium hypochlorite which is a disinfecting agent for treating drinking water at the household level. The households were informed and demonstrated about proper use of WaterGuard: to add one cap-full of the waterguard to a 20l jerrican containing the drinking water and allow it to stand 30 minutes after shaking it before starting drinking the treated water [16].

**Measured variable:** the outcome variable was diarrhea which is defined as the child´s experience of defecating feces for three and more than three times in a single day [1]. The occurrence of diarrhea was measured in terms of incidence. Hence, in the trial, incidence was computed by dividing number of new diarrheal episodes by person-weeks of observation [14].

**Sample size:** the sample size for this cluster randomized controlled trial was computed on the basis of 35% reduction in incidence of diarrhea in the intervention group compared to the control group [17]. The computed sample size was 816 under-five children (204 children in each group) with 80% power, 5% significance level, 95% confidence interval, 10% contingency for the expected non-response, and a design effect of four. Sample size for clusters was determined using the Hayes and Bennett method [18].

**Data collection:** baseline data on diarrhea related variables, data on occurrence of diarrhea and the bi-monthly use of the intervention materials were collected by data collectors, intervention providers and supervisors through a series of eight rounds. All the field workers such as data collectors, intervention providers and supervisors were recruited from the local area with education background of grade 10 completed for data collectors and intervention providers and high school graduates for supervisors. Moreover, all of them received training on proper methods of data collection for two days from the principal investigator. The main and secondary outcomes of this study were diarrhea and participants compliance with the interventions. Free residual chlorine tests were carried out and soap wrappers collected once every two weeks to measure the participants compliance to the intervention. Water samples for microbiological analysis were collected from 10% of the households in each of the four arms at the baseline in September 2018 and end point of the study period in January 2019. They were transported in an ice box within four hours of collection to the laboratory of Dire Dawa Water Supply and Sanitation Authority, where they were tested by membrane filtration technique for identification of *Escherichia coli*.

**Data analysis:** the data were entered into Epi- Data version 3.1 and analyzed after exporting them to STATA version 15.0. The analysis was primarily intended to compare the incidences among intervention and control arms. Crude and adjusted incidence rate ratios (cIRR and aIRR) with the accompanying confidence interval were calculated using generalized estimation equation with log link Poisson distribution [19].

**Ethical approval:** data were collected after obtaining ethical clearance from the National Research Ethics Review Committee (NRERC) in Addis Ababa. Before the commencement of the study, written consent was obtained from Dire Dawa Regional Health Bureau. Information about the study and its objectives was provided to mothers and caregivers and their written consent was obtained. Data collectors advised mothers and caregivers to seek care for diarrhea of their children at nearby health posts or health centers. Finally, households in the control arm were provided with waterguard and soap together with proper use of waterguard hand hygiene promotion after completion of the study to avoid any bias. The authors have no competing interests.

## Results

Of the 816 identified study participants, 812 consented to participate and completed the study with a response rate of 99.5%. In the follow-up period, data were obtained from 203, 204, 203 and 202 under-five children of the water treatment, handwashing, combined and control arms, respectively. The interventions and follow-up were implemented between October 2018 and January 2019. The number of households per cluster was at least 51. The mean family sizes per household were 4.24, 5.08, 5.76, and 5.91 for the four arms of water treatment, handwashing, combined and control arms, respectively. The median ages of the children and the mothers/caregivers were 36 (IQR: 24-48) months and 30 (IQR: 27-35) years for combined arm and 38 (IQR: 32-48) months and 28 (IQR: 25-30) years for control arm (Table 1). In the water treatment intervention, households in the intervention and control groups were comparable with respect to child sex, breastfeeding status, availability of soap in the home, availability of refuse disposal facilities, handwashing before preparing food, handwashing after use of toilet, handwashing after contact with child feces, narrowness or wideness of the household water storage container, owning a watch and television at the baseline. Pre-intervention two-week prevalence of diarrhea was 24.6% in the control group and 24.3% in the intervention group with no statistically significant difference in diarrhea rates. A manuscript on this intervention had already been published.

**Table 1:** characteristics of under-five children, their mothers/caregivers, and environmental factors at baseline in rural Dire Dawa, Eastern Ethiopia, 2019

Variables	Control arm n (%)	Combined arm n (%)
No of clusters	4	4
No of households	202	203
No of under-five children	202	203
Mean family size per household	5.91	5.76
Median age of under-five children	38 (IQR: 32-48)	36 (IQR: 24-48)
Median age of mothers/caregivers	28 (IQR: 25-30)	30 (IQR: 27-35)
**Child sex**		
Male	101 (50.0)	113 (55.7)
Female	101 (50.0)	90 (44.3)
**Latrine availability**		
No	170 (84.2)	60 (29.6)
Yes	32 (15.8)	143 (70.4)
**Availability of soap in the home**		
No	160 (79.2)	149 (73.4)
Yes	42 (20.8)	54 (26.6)
**Refuse disposal facility available**		
No	162 (80.2)	121 (59.6)
Yes	40 (19.8)	82 (40.4)
**Handwashing before preparing food**		
No	185 (91.6)	192 (94.6)
Yes	17 (8.4)	11 (5.4)
**Handwashing after use of toilet**		
No	176 (87.1)	157 (77.3)
Yes	26 (12.9)	46 (22.7)
**Father's occupation**		
Farmer	195 (96.5)	197 (97.0)
Other	7 (3.5)	6 (3.0)
**Water source**		
Unimproved	6 (3.0)	31 (15.3)
Improved	196 (97.0)	172 (84.7)
**Two-week prevalence of diarrhea**		
No	153 (75.7)	162 (79.8)
Yes	49 (24.3)	41 (20.2)

The handwashing intervention and control groups were similar in regard to child sex, mother's education, availability of soap in the home, handwashing before preparing food, handwashing after use of toilet, narrowness or wideness of the household water storage container, availability of refuse disposal facility, presence of watch and television at the baseline. The two-week prevalence of diarrhea at the baseline was 25.5% in the control group and 24.3% in the intervention group and no statistically significant difference was detected between the two groups. Similarly, a manuscript on this intervention had also been published. The combined intervention and control groups were similar in regard to sex of the children, fathers´ occupation, mothers´/caregivers´ handwashing practices before preparing food and availability of soap in the home at the baseline. The two-week prevalence of diarrhea at the pre-intervention phase was also comparable in the control and intervention households (Table 1). Eight households (4.0%) from the control group, 11 households (5.4%) from the water treatment group, 14 households (6.9%) from the handwashing group, and 15 households (7.4%) from the combined group were treating their drinking water using different methods at the baseline.

**Figure 2:**
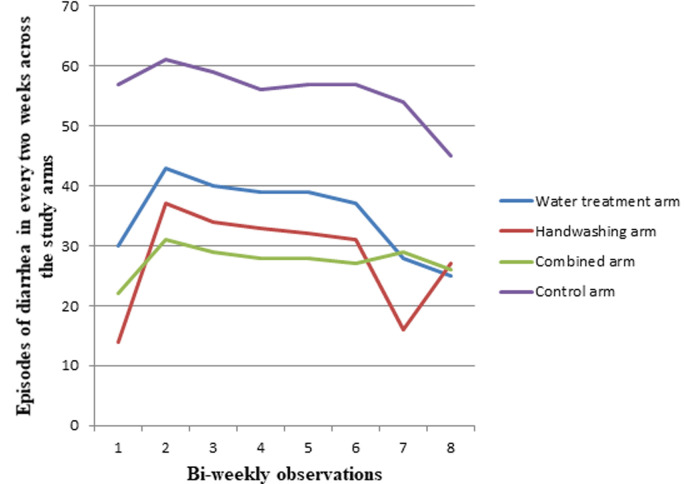
total bi-weekly episodes of diarrhea observed in each arm of the study, rural Dire Dawa, Eastern Ethiopia, 2019

**Incidence of diarrhea:** in the water treatment arm (WT) a total of 281 episodes of diarrhea were recorded in the 16 week follow up period (8.7 episodes per 100 person-weeks of observation) and in the handwashing arm (HW) a total of 224 episodes of diarrhea were recorded (6.9 episodes per 100 person-weeks of observation). Similarly, in the combined treatment arm (Co) a total of 220 episodes of diarrhea were recorded (6.8 episodes per 100 person-weeks of observation). However, in the control arm (C) a total of 446 episodes of diarrhea were recorded, with the resulting greater episodes unlike the intervention arms (13.8 episodes per 100 person-weeks of observation). The total episodes of diarrhea every two weeks versus weeks of observation across each arm of the study are presented in Figure 2. No harm was detected in this interventional study. In the water treatment arm, after adjusting for sex of the child, presence of refuse disposal facility, availability of latrine, availability of handwashing facility and presence of soap in the home, under-five children in the intervention group had lower risk of diarrhea (adjusted IRR = 0.64, 95% CI: 0.57 - 0.73). An overall 36% reduction in incidence of diarrhea was observed in the intervention group compared to the control group. In the handwashing arm, under-five children had lower risk of diarrhea (adjusted IRR = 0.588, 95% CI: 0.53 - 0.65) having accounted for sex of the child, presence of refuse disposal facility, availability of latrine, the household water source, and water storage container. Hence, a 41% reduction in diarrheal incidence was observed in the intervention group. Similarly, in the combined arm, under-five children had lower risk of diarrhea (adjusted IRR = 0.585, 95% CI: 0.53 - 0.65) having adjusted for sex of the child, mother's education, father's occupation, family size, availability of latrine and availability of refuse disposal facility. There was a 41% overall reduction in incidence of diarrhea in the intervention group in comparison to the control group. This 41% reduction in incidence of diarrhea in both the combined arm and the handwashing arm indicates that water chlorination did not confer additional benefits in those households receiving the combined intervention (Table 2).

**Table 2:** multivariable analysis of the effect of combined intervention on the incidence of diarrhea among children under the age of five years in rural Dire Dawa, Eastern Ethiopia, 2019

Factors	Crude IRR (95% CI)	Adjusted IRR (95% CI)	p-value
**Combined intervention**	0.586 (0.53 - 0.65)	0.585 (0.53 - 0.65)	< 0.001
Control	1	1	
**Child age**	1.00 (0.99 - 1.00)	1.00 (0.99 -1.00)	0.865
**Child sex**			
Female	0.99 (0.91 - 1.08)	0.99 (0.91 - 1.08)	0.887
Male	1	1	
**Mother's education**			
Primary and above	1.00 (0.92 - 1.10)	0.99 (0.90 - 1.10)	0.946
No formal education	1	1	
**Father's occupation**			
Miscellaneous	0.99 (0.77 - 1.29)	0.99 (0.77 - 1.28)	0.941
Farmer	1	1	
**Family size**	0.99 (0.98 - 1.02)	0.99 (0.98 - 1.02)	0.882
**Presence of latrine**			
Yes	1.01 (0.92 - 1.11)	1.00 (0.91 -1.10)	0.955
No	1	1	
**Presence of refuse disposal facility**			
Yes	0.98 (0.90 - 1.07)	0.98 (0.89 - 1.07)	0.634
No	1	1	

**Microbial water quality:** in the combined water treatment and handwashing arm, 85.0 and 80.0% of the households had *Escherichia coli* in the water samples of the intervention and control groups, respectively, at base line. *Escherichia coli* count differences between intervention and control households (p = 0.552) at the start of the study were not significantly different. However, at the end of the study 40.0% of the water samples from the intervention households and 85.0% of the samples from the control households showed contamination with a statistically significant mean *Escherichia coli* count difference (p = 0.030) (Table 3).

**Table 3:** microbial water quality between intervention and control households at the beginning and end of the intervention in rural Dire Dawa, Eastern Ethiopia, 2019

Water treatment and handwashing combined arm			
*Escherichia coli* in drinking water	Intervention households n (%)	Control households n (%)	p-value
Base line	17 (85.0)	16 (80.0)	0.552
At end of the study	8 (40.0)	17 (85.0)	0.030

**Adherence to the interventions:** in the water treatment arm, data collectors measured free residual chlorine concentrations every two-week schedule anytime in the two-week period and the measurement of free residual chlorine on every two-week basis continued until the end of the study. In 81.28% of the water samples tested from the intervention households, free residual chlorine of > 0.2mg/l was found. In the handwashing arm, data collectors regularly collected wrappers of the soap bars of the previous two weeks from each participating household before giving each of them an additional bar. They collected soap wrappers from 78.35% of the 204 households during the 16-weeks study period. In the combined arm, data collectors measured the free residual chlorine every two weeks and collected soap wrappers in exchange for additional soaps every two weeks. The combined adherence was 82.66%.

## Discussion

In this study we assessed the effectiveness of WASH intervention methods in reducing diarrhea among under-five children using community-based cluster randomized controlled trial. In all, children in households employing either drinking water disinfection by chlorination or handwashing with soap or the combined of the two showed lesser diarrheal cases than children in households practicing usual habits of handwashing and water handling. The three intervention arms collectively lacked the following basic facilities: 48.9%, 75.2%, 85.6%, 79.5%, 60.8%, 90.2%, 78.7%, and 27.0% of the households had no latrine, refuse disposal facilities, handwashing facilities, soap, radio, television, concrete floor, and improved water source, respectively. In spite of all this difficulties, our study was successful in achieving these significant reductions in incidence of diarrhea among children under five years of age. A better reduction in incidence of diarrhea was achieved in children who received the combined intervention (41%) than those who received the single intervention of household water treatment (36%). However, children receiving the combined intervention showed no greater reduction in incidence of diarrhea than children in households who received the single intervention of handwashing (41%). Hence, combining the two interventions had no better effect than the single intervention. In this study, the combination of household chlorination with sodium hypochlorite and handwashing with soap reduced diarrhea significantly; with a 41% lower diarrheal incidence in intervention households than in control households. This reduction was lower than in a study in Pakistan (55%) [11]. However, the combined intervention failed to reduce the incidence of diarrhea more than the single intervention of handwashing. These results corroborate studies in Pakistan [11], Afghanistan [10], Kenya [12] and Bangladesh [13].

Furthermore, a systematic review and meta-analysis on WASH interventions reported that multiple interventions consisting of combined water, sanitation, and hygiene measures were not more effective than interventions with a single focus [4]. This anomaly may be due to the difficulty of maximizing the effectiveness of handwashing promotion and water disinfection with sodium hypochlorite in four months. The difficulty of changing old customs and traditions of not chlorinating drinking water and not washing hands with soap further indicates that the uptake of new hygiene related behaviors take more time [20]. Another explanation could be that some households preferred to adopt only one of the two interventions because of time constraints or the perception that one intervention was sufficient to prevent diarrhea. The microbiological quality of drinking water in households receiving water treatment, the handwashing and the combined water treatment and handwashing arms were significantly higher (p < 0.05) than in households in the control arm at the end point of the study period. Furthermore, the microbiological qualities of the drinking water in the single and combined interventions were comparable at the end of the study. This finding is in agreement with similar studies in Kersa District [21] and Jigjiga District [17]. Moreover, this study was conducted in the dry season when fecal contamination of the environment is less likely to occur than during rainy season. For instance, a study in Tanzania showed that enterotoxigenic Escherichia coli and Giardia lamblia were most prevalent in the rainy season [22]. Hence, conducting the intervention study in the dry season helped us to show the effect of the interventions in the absence of environmental impact. In this study, 80.0% of the households in the water treatment arm, 81.9% of the households in the handwashing arm, and 94.6% of the households in the combined arm, respectively, stored their drinking water in narrow-necked containers. Further, water stored in wide-necked containers is more likely to become contaminated [23]. This might synergistically be complemented with the interventions to achieve these significant reductions in incidence of diarrhea among children younger than five years.

**Limitations:** the following limitations of the study may have influenced the results. First, this is an open trial instead of a blinded type trial because we were unable to employ blinding due to the odor and taste of sodium hypochlorite. Second, household visits by the data collectors once every two weeks to record the occurrence or non-occurrence of diarrhea among children younger than five years of age may have resulted in recall bias. We tried to minimize recall bias by properly training data collectors by instructing them to carefully ask mothers/caregivers about the day when the diarrhea started and when it ended. Third, in this study the intervention materials were provided for free to the study population, possibly resulting in courtesy bias and Hawthorne effect (observer effect). We used independent intervention providers to provide the intervention materials (bottles of WaterGuard and bars of soaps) to reduce this effect. Thus, the data collectors collected information only on the episodes of diarrhea during each of the two-week periods without providing intervention materials. Fourth, due to the constraints of money and time, the intervention study was carried out only for four months without considering seasonality issues. Recruiting data collectors, intervention providers and supervisors from the local population, involving community leaders during randomization of clusters, and motivating the intervention households to continually use the provided intervention materials were some of the strengths of this study.

## Conclusion

In conclusion, independent implementation of household water disinfection using sodium hypochlorite and household handwashing with hand hygiene promotion reduced episodes of diarrhea among children under-five years of age in rural Dire Dawa. However, their combination was no more effective than the individual ones. Thus, we recommend implementing either household water disinfection using sodium hypochlorite or household handwashing with hand hygiene promotion independently at large scale to vulnerable population would help to reduce diarrheal morbidity considerably till the sixth sustainable development goal (SDG-6) is ensured. Moreover, we recommend the use of longer study period to address seasonality issues.

### What is known about this topic

- *Household chlorination was effective in reducing diarrhea in children compared to non-intervention;*
- *Handwashing was effective in reducing diarrhea in children;*
- *The combination of household chlorination and handwashing is expected to be more effective than the single intervention of either of the two*.

### What this study adds

- *All the independent interventions were effective in reducing diarrheal disease among children under the age of five years. However, handwashing was a little bit more effective than household chlorination;*
- *Their combination was more effective than the single intervention of household chlorination;*
- *Their combination was no more effective than the single intervention of handwashing*.
